# SurvGRN: a multi-feature fusion framework for bladder cancer survival prediction

**DOI:** 10.1097/JS9.0000000000004874

**Published:** 2026-06-02

**Authors:** Qiangjian Zhang, Na Zhao, Kai Song, Xun Ye, Hongxin Xiang, ZhiWei Zhao, Li Qi, Qing Zhang, Rui Guo, Junwei He, Jiaxin Liang, Shi Fu, Haifeng Wang, Xiaogang Li, Yingxia Wang, Yingying Pu, Jian Wang, Chunming Guo

**Affiliations:** a School of Software and AI, Yunnan University, Kunming, Yunnan, China; b Yunnan Key Laboratory of Cell Metabolism and Disease, and Center for Life Sciences, School of Life Sciences, Yunnan University, Kunming, Yunnan, China; c College of Computer Science and Electronic Engineering, Hunan University, Changsha, China; d Department of Urology, The Second Affiliated Hospital of Kunming Medical University, Kunming, China; e Department of General Surgery, Affiliated Hospital of Yunnan University, Kunming, Yunnan, China; f The Department of Pathology of First affiliated Hospital, Kunming Medical University, Kunming, Yunnan, China; g The State Key Laboratory Breeding Base of Basic Science of Stomatology & Key Laboratory of Oral Biomedicine Ministry of Education, School & Hospital of Stomatology, Medical Research Institute, Wuhan University, China; h College of Information Engineering and Automation, Kunming University of Science and Technology, Kunming, China

**Keywords:** bladder cancer, gated residual networks, multi-feature fusion, patient survival outcomes prediction

## Abstract

Bladder cancer survival outcomes exhibit significant heterogeneity, influenced by multifaceted factors. While digital pathology-based survival models leveraging artificial intelligence show promise, they often overlook complementary data sources. Conversely, imaging lacks cellular detail, and genomics/proteomics entail complexity and cost. To integrate multidimensional data for enhanced survival prediction, we propose SurvGRN, a multi-feature fusion framework. SurvGRN synergistically combines clinical variables, transcriptomics, and digital pathology slides using a gated residual network architecture. Pathological features are extracted via multiple instance learning, while clinical and transcriptomic data are processed as static inputs. These features are dynamically fused using a long short-term memory (LSTM) network for comprehensive survival risk assessment. Evaluated on 400 bladder cancer patients, SurvGRN significantly outperformed existing methods: improving the C-index by 12.6% over DeepMISL; 20.6% and 7.1% over graph-based models (DeepGraphConv and Patch-GCN); and 5.4% and 4.0% over attention-based approaches (Surformer and HVTSurv). Ablation studies confirmed the contributions of pathology features (extracted via ResNet-50 pre-trained on bladder tissue), clinical/transcriptomic data, and the LSTM fusion. SurvGRN also enabled significant stratification of patients into distinct risk cohorts. This work demonstrates that holistic integration of multi-source data through tailored fusion architectures substantially improves bladder cancer survival prediction.

## Introduction

As a common urological malignancy, the incidence of bladder cancer has been steadily increasing^[^1^]^. In 2020, there were nearly 600 000 new cases and approximately 210 000 associated deaths worldwide^[^2^]^. The prognosis of bladder cancer is closely related to a variety of factors, such as the stage of the tumor^[^3^]^, lymph nodes^[^4^]^, the patient’s transcriptional profile^[^5^]^, and overall health status^[^6^]^.

HIGHLIGHTS

- We proposed SurvGRN, a novel survival analysis model for bladder cancer patients that fuses clinical, transcriptomic, and digital pathology slide data.
- SurvGRN adopts a multi-instance learning framework with gated residual networks and long short-term memory (LSTM) network, enhancing feature extraction and survival risk prediction.
- On a multi-feature dataset of 400 bladder cancer patients, SurvGRN achieved a C-index of 0.614, outperforming existing methods such as DeepMISL, Patch-GCN, and Surformer.
- Ablation studies confirm the effectiveness of integrating multi-feature data and model components, including the LSTM structure and static feature augmentation.
- SurvGRN absorbed key prognostic biomarkers, such as ARID1A, KDM6A, CDKN2A, and PTEN, offering insights for personalized prognosis and treatment.

In recent years, the rapid development of medical image digitization and artificial intelligence (AI) technology has led to the widespread application of AI across a growing number of fields^[^7–11^]^. There have been numerous examples of successful applications of machine learning, particularly those leveraging Convolutional Neural Networks (CNNs) based on Whole Slide Images (WSIs) to analyze skin cancer^[^12,13^]^, breast cancer^[^14,15^]^, lung cancer^[^16,17^]^, and prostate tumors^[^18–20^]^. Most existing CNNs for WSI analysis primarily focus on pathological diagnosis, including tumor cell detection and classification^[^21–23^]^, survival analysis^[^24–26^]^, and grade classification^[^27^]^.

With the development of new technologies, multi-omics fusion techniques have received widespread attention in cancer research. Studies have shown that by integrating different levels of histologic data, the complex biological mechanisms of cancer can be better understood, and the predictive accuracy of prognostic models can be improved^[^28–33^]^. Multi-omics fusion aims to integrate information from different data sources, such as genomics, transcriptomics, proteomics, and metabolomics, to leverage richer biological in formation for analysis^[^28–33^]^. This approach provides significant potential for utilizing the wealth of information generated by medical research and clinical practice. By combining digital pathology image data with molecular information from genomics and other bioinformatics, multi-omics fusion can enhance prognostic analysis for patients^[^34–36^]^. This multidimensional integration of information allows models to capture biological changes at different levels, more accurately reflecting the complexity and heterogeneity of the disease. For example, genomics data can reveal genetic mutations, transcriptomics data can show gene expression levels, and proteomics and metabolomics data can provide information about dynamic changes in proteins and metabolites. Integrating these data enables a more comprehensive understanding of the onset and progression of the disease, thereby improving the accuracy of prognostic analysis.

However, despite the significant advantages of multi-omics fusion, its implementation faces many challenges. First, data from different sources often vary in size, format, and noise levels, making effective preprocessing and standardization a critical challenge. Second, the integration of different types of histologic data requires the development of new computational methods and algorithms. Additionally, the high dimensionality and het erogeneity of multi-omics data increase the complexity and computational cost of model training. The main difficulties faced in multi-omics fusion include the heterogeneity and complexity of data, the need for computational resources, and the lack of standardized methods. Data from different plat forms and technologies may differ significantly in quality and feature distribution. Directly integrating such data can introduce noise and bias, while the high-dimensionality of multi-omics data requires substantial computational resources and storage space, imposing higher demands on processing power and algorithmic efficiency.

Most of the existing bladder cancer prognostic analysis models rely on a single type of data, such as using only genomics data or medical imaging data. These methods, while providing valuable predictive information to some extent, tend to ignore the complexity and multilevel biology of cancer. Models based on a single data source are susceptible to data noise and bias, resulting in suboptimal predictive accuracy and robustness. In addition, the lack of comprehensive integration of clinical and transcriptomic information limits the effectiveness and generalizability of the models in practical clinical applications. Therefore, the introduction of clinical information and transcriptomic information is of great significance for enhancing the performance of bladder cancer prognostic analysis models. Clinical information, such as the patient’s age, gender, and medical history, can provide valuable clues about the patient’s overall health status and disease progression, thereby improving the predictive ability of the model. Similarly, transcriptomic information reveals dynamic changes in gene expression, reflecting the biological behavior of tumor cells and the molecular mechanisms of the disease. When combined with genomic information, transcriptomic data provide more comprehensive insights at the molecular level.

In summary, this paper proposes SurvGRN, a model for prognostic analysis of bladder cancer patients. This model integrates information sources such as medical imaging data and multi-omics genetics information. It extracts input features using a static variable extractor and utilizes a multi-instance learning (MIL) method to process slide information, ultimately performing survival analysis. The integration of these information sources provide a deeper and more comprehensive understanding of bladder cancer, thereby improving the accuracy and reliability of the model. Although multi-omics fusion faces challenges such as data heterogeneity, complexity, and computational re source requirements, it has great potential and is expected to contribute to the realization of individualized medicine and precision medicine.

## Result

**Figure 1.**
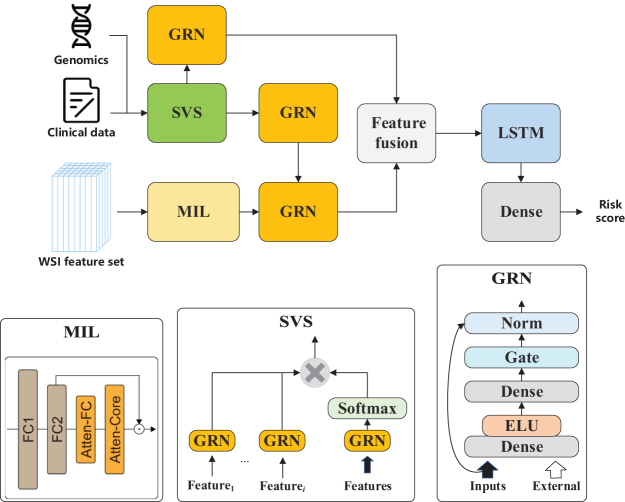
Structure of SurvGRN: a multi-feature fusion-based survival analysis model for bladder cancer patients.

In this paper, we designed a novel survival analysis model (SurvGRN) for bladder cancer patients, whose overall architecture is shown in Figure 1. The model extracts clinical information, transcriptomics information, and digital pathology slide information from these patients and fuses all this information together for analysis. To verify the model’s validity and robustness, clinical data, digital pathology slide data, and genomics data containing 400 bladder cancer patients were collected to verify the validity and robustness of the model. The 400 patients were divided into a training set and a test set, each containing 50% of the data, and the performance of the model was evaluated using a consistency index (C-index). After being trained on the training set, the SurvGRN achieved a C-index value of 0.614 on the test set, which represents a 12.6% improvement compared to DeepMISL^[^37^]^ (Deep Multi-Instance Survival Learning, a deep learning model based on MIL) (C-index value of 0.536), DeepGraphConv improved by 20.6% (C-index of 0.499), Patch-GCN improved by 7.1% (C-index of 0.562), Surformer improved by 5.4% (C-index of 0.571), and HVTSurv improved by 4.0% (C-index of 0.579). In addition, this paper explores the potential and value of the model through ablation experiments, identification of key impact factors, and survival analysis evaluations. The SurvGRN model proposed in this paper demonstrates high performance and robustness in survival analysis of bladder cancer patients, highlighting its significant potential for clinical application. It provides a new research direction and technical support for advancing precision medicine in bladder cancer.

### Model structure

In this paper, we use the pre-trained ResNet-50 backbone network to extract the features of digitized bladder cancer slices, process these features using the multiple instance learning (MIL) method^[^38^]^, and combine this approach with the Temporal Fusion Transformer^[^39^]^, a framework for analyzing time series data. Based on these components, we proposed a survival analysis model for bladder cancer patients based on multi-feature fusion, named SurvGRN.

The input of SurvGRN consists mainly of clinical information, transcriptomics information, and digital pathology section information. The input data include three parts: the clinical data, which consist of the patient’s gender, the patient’s tumor–node–metastasis (TNM) staging status, the TNM staging tumor spreading status, whether or not drug treatment was administered, whether or not radiation treatment was given, tumor location information, and the patient’s diagnostic time; the transcriptomic information, which consists of the Yang oncogenes, oncogenes, and typing indexes that were constructed; and the digital pathology slide information, which was extracted using a pre-trained ResNet-50 model to characterize the digitized slices of bladder cancer. The purpose of introducing these data is reflected in the different distribution characteristics of each type of information and its impact on the prediction results as follows:

Gender: There are significant differences in the incidence and prognosis of bladder cancer by gender. The incidence of bladder cancer is usually higher in male patients than in females, but the prognosis for female patients may be poorer.

TNM staging status and tumor spread: TNM staging reflects the severity and spread of the tumor, and patients with higher staging usually have a poorer prognosis, so this is a key predictor.

Treatment information: The presence or absence of drug therapy and radiation therapy has a significant impact on patient survival. Patients who have received these treatments may have a different survival prognosis.

Tumor location information: Tumors in different locations may have different growth characteristics and prognosis.

Time to diagnosis: Time to diagnosis can help establish a patient’s treatment history and survival timeline, affecting the timeliness and accuracy of predictive models.

Oncogenes and oncogenes: The expression levels of these genes directly affect tumor growth and metastasis. Differences in the expression of these genes between different patients can provide prognostic information.

Staging indicators: Classification of tumors based on molecular features can help identify groups of patients with different prognoses. Fusion of multimodal data can improve the predictive power and stability of models. The combination of multiple data sources can reduce the limitations of a single data source and improve the reliability and accuracy of prediction results. Introducing these data not only provides their independent information alone but also enables more accurate individualized prognosis prediction through fusion analysis.

Table 1 of data types is shown below: The SurvGRN model usually inputs numerical data directly into the model for processing, while category-type data need to be preprocessed. In this paper, we refer to word embedding and use the Embedding method to preprocess the category-type data, and each of the category-type data uses one word embedding each to generate the corresponding word embedding vectors. The numerical data and the word embedding vectors converted from the category-type data are then converted into multiple vector features of the same size as static variable features using a mapping network.

**Table 1:** Data types of clinical and transcriptomics information.

Category	Name	Data type
Clinical data	Gender of the patient	Categorical
	TNM staging of the patient	Categorical
	TNM stage of tumor spread	Categorical
	Medication treatment	Categorical
	Radiation therapy	Categorical
	Tumor location	Categorical
	Diagnosis time	Numeric
Oncogene	FGFR3	Numeric
	E2F3	Numeric
	FGFR1	Numeric
	EGFR	Numeric
	KRT7	Numeric
Tumor suppressor gene	RUNX3	Numeric
	ARID1A	Numeric
	CDKN2A	Numeric
	PTEN	Numeric
	TP53	Numeric
	EP300	Numeric
	KDM6A	Numeric
	KDM6B	Numeric
	KMT2D	Numeric
	KMT2C	Numeric
Staging indicators	PPARG	Numeric
	UPK3A	Numeric
	KRT5	Numeric

The main process of SurvGRN, a survival analysis model for bladder cancer patients based on multi-feature fusion, is as follows:

First, the SurvGRN model uses a static variable extractor (SVS) to process patients’ static variable features, such as age, gender, and clinical stage. The SVS consists of multiple gated residual networks (GRNs), and each GRN is responsible for extracting the static variable features for a specific dimension. The output results from the SVS are used in two modules: one part is used for feature fusion, and the other part serves as additional input features to enhance the feature representation of digital pathology slides. To accomplish this, the SurvGRN model applies two separate GRNs to the output results of the SVS separately: one GRN for extracting the static variable features required for feature fusion, and the other GRN for extracting the additional input features.

Second, the SurvGRN model uses MIL to process the digital pathology slide information of bladder cancer. MIL enables the model to learn the overall features of the patient from multiple slide images without requiring labels for each individual slide image. The output result of MIL is fed into another GRN, which is fused with the additional input features from SVS to obtain the final digital pathology slide feature representation.

Finally, the SurvGRN model splices the outputs of SVS and MIL to form a comprehensive feature vector. This feature vector is fed into a long short-term memory (LSTM) network for further feature extraction; the output of the LSTM is fed into a linear layer to compute the patient’s survival risk value for survival analysis.

The model consists of three main modules: MIL, GRN, and static variable extractor network. It analyzes and processes the patient’s clinical information, transcriptomics information, and digital pathology slide information, and finally outputs the patient’s survival risk.

### Model comparison

The consistency index (C-index), which assesses the degree to which predicted values agree with observed values, is used in the evaluation of many survival analysis models^[^40–42^]^ and therefore, we use the C-index to evaluate the performance of the SurvGRN model.

In this part of the experiments, we use the ResNet-50 model trained with a bladder cancer image dataset (TCGA-BLCA) constructed from 50 digital pathology slides of bladder cancer and extract the features of the bladder cancer pathology slides. Subsequently, we randomly divide the 400 bladder cancer patients with TNM staging of II–IV into the training set and the test set, and perform a 10-fold cross-validation. We compared the proposed SurvGRN model with several state-of-the-art survival analysis methods, and the models all used the same loss function and training hyperparameters to ensure consistent performance across the models.

The C-index of each model in the bladder cancer patient cohort is given in Table 2, where our SurvGRN model improves by 12.6% compared to the MIL-based method (DeepMISL); by 20.6% and 7.1% compared to the graph convolutional network-based methods (DeepGraphConv and Patch-GCN); and by 20.6% and 7.1% compared to the self-attention-based methods (Surformer and HVTSurv), which improved by 5.4% and 4.0%, respectively.

**Table 2:** Comparison of results with other methods.

Methods	C-index
DeepMISL^[^37^]^	0.536 ± 0.038
DeepGraphConv^[^43^]^	0.499 ± 0.057
Patch-GCN^[^44^]^	0.562 ± 0.039
Surformer^[^45^]^	0.571 ± 0.032
HVTSurv^[^46^]^	0.579 ± 0.019
**SurvGRN (ours)**	**0.614 ± 0.081**

Compared with DeepMISL and DeepAttenMISL, our model is also based on the MIL method for extracting digital pathology slides of bladder cancer and introduces a gated residual module. However, compared with the above two models, SurvGRN incorporates multi-omics data and utilizes clinical data, digital pathology slide data, and genomics data for survival analysis, resulting in greater enhancement, which further confirms the advantage of multi-omics data.

### Ablation experiment

From the previous section, it is evident that the SurvGRN model incorporates clinical information, transcriptomics information, and digital pathology slide features to predict the survival risk value of patients. To evaluate the effectiveness of each module of the model, this paper splits and combines the modules of SurvGRN, assessing the performance of each structure using a dataset of 400 bladder cancer patients.

**Figure 2.**
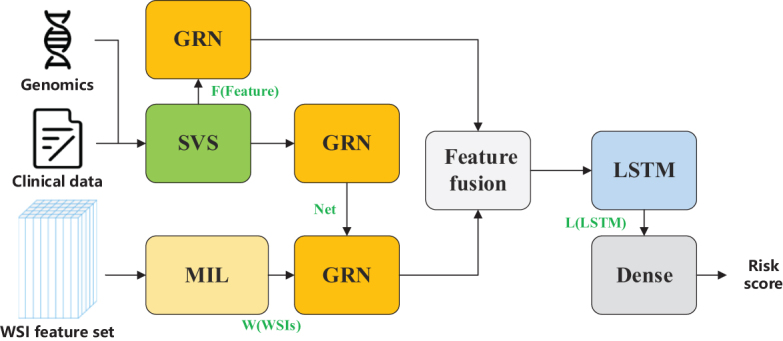
Schematic structure of the ablation experiment model.

Our primary hypothesis is that the accuracy of survival analysis can be improved by utilizing three types of features, namely, features of digital pathology slides, clinical information, and transcriptomics information, as well as the LSTM structure to capture the features after fusion. Table 3 and Figure 2 outline the 10 model structures compared in this study, where W represents digital pathology slide features, F refers to the two types of static features (clinical and transcriptomics data), L denotes the LSTM structure, and Net denotes the use of additional input features. The combination W + F + L + Net represents our proposed SurvGRN model, while the remaining architectures serve as benchmarks for comparison.

**Table 3:** Structure of the ablation experiment model.

Model number	Model structure	Description
1	F	Utilize static features for survival analysis
2	F + L	Utilize static features combined with LSTM for survival analysis
3	W	Utilize features from digital pathology slides for survival analysis
4	W + Net	Utilize features from digital pathology slides, and use static features as additional input for survival analysis
5	W + F	Utilize features from digital pathology slides and static features for survival analysis
6	W + F + Net	Utilize features from digital pathology slides and static features, and use static features as additional input for survival analysis
7	W + L	Utilize features from digital pathology slides combined with LSTM for survival analysis
8	W + L + Net	Utilize features from digital pathology slides, combined with LSTM, and use static features as additional input for survival analysis
9	W + F + L	Utilize features from digital pathology slides and static features, combined with LSTM for survival analysis
10	W + F + L + Net	The SurvGRN model proposed in this paper

Table 4 shows the performance of each module on the training and test sets. Notably, the SurvGRN model W + F + L + Net performs optimally among all models, achieving a C-index of 0.614, which significantly outperforms models using only a single feature or no LSTM structure.

**Table 4:** C-index results of ablation experiments on the test set.

Model structure	C-index	Model structure	C-index
F	0.602	W + L	0.596
W	0.594	W + F + Net	0.607
F + L	0.595	W + L + Net	0.610
W + Net	0.609	W + F + L	0.611
W + F	0.605	**SurvGRN**	**0.614**

We first analyzed the impact of incorporating static features as additional inputs. SurvGRN improved by 0.003 compared to W + F + L, W + L + Net improved by 0.014 compared to W + L, and W + F + Net improved by 0.002 compared to W + F, demonstrating that the use of static features as additional inputs can improve the performance of the model.

Next, we evaluated the efficacy of static features independently. SurvGRN improved by 0.004 compared to W + L + Net; W + F + Net decreased by 0.002 compared to W + Net; and W + F improved by 0.002 compared to W, which indicates that the static features in SurvGRN can improve the final performance of the model.

Finally, we evaluated the impact of the LSTM structure. SurvGRN improved by 0.007 compared to W + F + Net, W + L + Net decreased by 0.001 compared to W + Net, and F + L improved by 0.007 compared to F, which indicates that LSTM can handle the fused features as well as static features effectively, and it can improve the performance of the model.

For the features of digital pathology slides, W + F + L demonstrated an improvement of 0.006, and W + F improved by 0.003 compared to F + L, indicating that these features contribute significantly to improving the final performance of the model.

Overall, the ablation experiments verified the validity and superiority of our proposed SurvGRN model. These results also highlighted the relative contributions of different features and modules to the survival analysis, offering valuable insights for future research directions.

Notably, static features (genomic features) hold substantial value in bladder cancer prognostic analysis, as they can provide rich biological information and thus improve the predictive accuracy of the model. The performance of the model is further improved by combining digital pathology slide features, network features, and LSTM. However, the extent of this improvement depends on the specific data characteristics and the model's architecture. By integrating the SurvGRN model with multiple information sources, the optimal prediction performance is finally achieved, demonstrating the great potential of multi-omics fusion in individualized and precision medicine.

### Key impact factor

**Figure 3.**
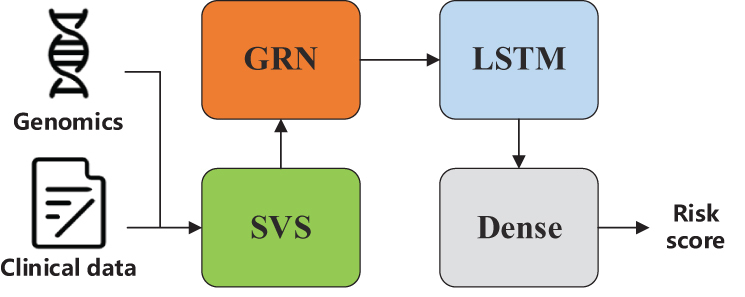
Structural diagram of the model for extracting key impact factors.

The SurvGRN model proposed in this paper enables survival analysis while assessing the degree of influence of the key factors on model predictions once the model parameters are determined. To minimize the potential interference that may be caused by the features extracted from the digital pathology slide, the module for processing the features of the digital pathology slide is therefore removed in this paper, and the structure of the model is shown in Figure 3.

**Figure 4.**
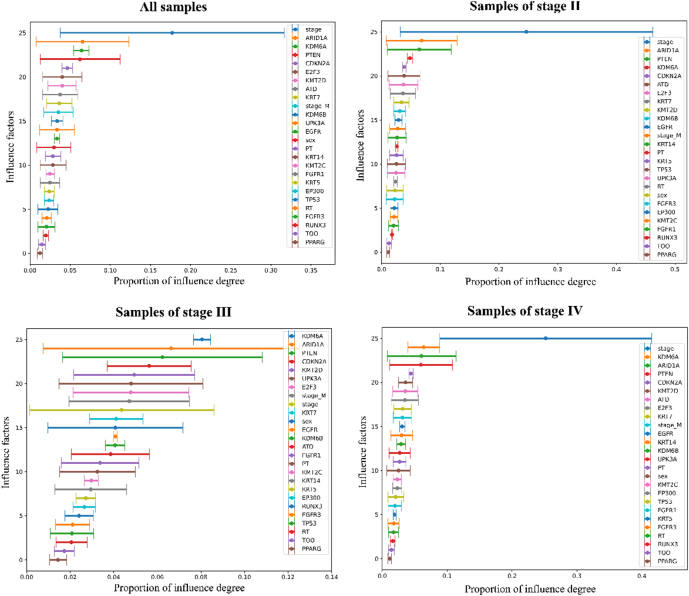
Results of the ranking of the degree of influence of the factors. (A) Impact factor ranking results based on the full sample; (B) impact factor ranking results based on the stage II sample; (C) impact factor ranking results based on the stage III sample; and (D) impact factor ranking results based on the stage IV sample.

Ten experiments were conducted randomly, and the mean value of the C-index on the training set was 0.727. Using the trained model, the clinical data and transcriptomic data of 200 bladder cancer patients were calculated by the model, and the relative influence degree of each influencing factor was outputted using the SVS module. Then, the mean value and the standard deviation of all the samples were calculated, and the mean value and the standard deviation of the patients who were at the TNM stage II and stage IV were calculated, respectively. Subsequently, the mean and standard deviation of all the samples were calculated again, along with the mean and standard deviation of patients with TNM stage II and stage IV, respectively. In this paper, we take the factors with the top 10 influence degrees as the key influencing factors. The distribution of the influence degree of the factors in each sample is shown in Figure 4.

Among all the samples, the key influencing factors are stage, ARID1A, KDM6A, PTEN, CDKN2A, E2F3, KMT2D, ATD (age at diagnosis), KRT7, and stage M. Of these, stage, ATD (age at diagnosis), and stage M are clinical features; ARID1A, KDM6A, PTEN, CDKN2A, and KMT2D are classified as oncogenes, and E2F3 and KRT7 are classified as proto-oncogenes.

The findings revealed that mutations or aberrant expression of these genes significantly influence bladder cancer progression across different stages.

In the experiment to identify key influencing factors, the features with the greatest influence on the model prediction were stage, ARID1A, KDM6A, PTEN, and CDKN2A. Amongthese, stage represents the TNM staging of bladder cancer tumors, determined based on the tumor information from the pathological sections. ARID1A, KDM6A, CDKN2A, and PTEN are the results of oncogene, which can restrict cell proliferation and play a very important negative role in the regulation of cell growth. ARID1A is involved in transcription, DNA damage repair, cell cycle control, and other processes. Mutations or deletions in ARID1A have been identified in various cancers, including bladder, ovarian, and pancreatic cancers, and are strongly associated with tumorigenesis and progression^[^47^]^. KDM6A can activate H3K27me3 modified genes, which are critical for processes such as cellular differentiation, proliferation, and apoptosis. Mutations or deletions in KDM6A are found in a variety of cancers, including bladder cancer, renal cancer, and esophageal cancer^[^48^]^. CDKN2A can prevent cells from entering the S phase from the G1 phase, and its mutation or deletion is found in a variety of cancers, including melanoma, pancreatic cancer, and lung cancer^[^49^]^. PTEN can regulate the processes of cell growth, apoptosis, migration, and metabolism, and its mutations or deletions are found in a variety of cancers, including breast cancer, thyroid cancer, kidney cancer, and melanoma^[^50^]^.

### Pre-training ResNet-50 on different datasets

In this section, we explore the impact of training the ResNet-50 model on different datasets on the final performance of the SurvGRN model, using C-index metrics, survival curves, and evaluation criteria.

We pre-trained the ResNet-50 model using three datasets: the ImageNet dataset^[^51^]^, the human colorectal cancer and healthy tissue image dataset^[^52^]^, and the bladder cancer image dataset. Features were extracted from bladder cancer pathology slices using these pre-trained models, named ImageNet, NCT-CRC-HE, and TCGA-BLCA, respectively. Subsequently, the 400 bladder cancer patient cases were randomly divided into training and test sets, and a 10-fold cross-validation was performed.

Table 5 shows that the model pre-trained with the TCGA-BLCA dataset produced the best results, followed by the model pre-trained with NCT-CRC-HE.

**Table 5:** C-index results for various pre-training datasets.

Pre-training dataset	Training set	Test set
ImageNet	0.73 ± 0.077	0.60 ± 0.077
NCT-CRC-HE	0.75 ± 0.089	0.60 ± 0.089
TCGA-BLCA	0.76 ± 0.077	0.61 ± 0.081

**Figure 5.**
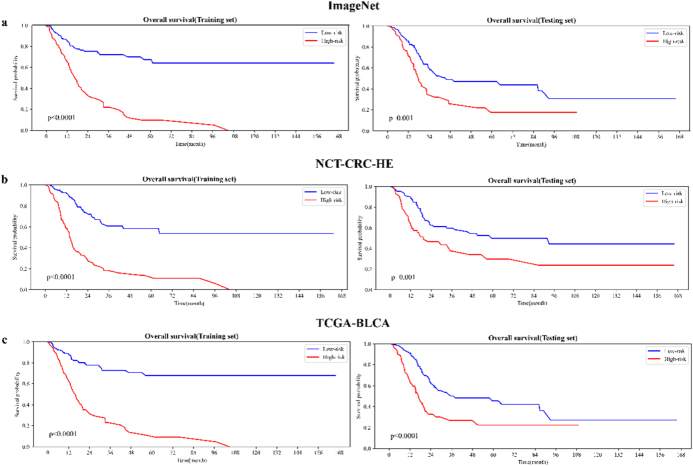
Classification of bladder cancer patients according to model-predicted survival risk values. (A) Patient subgroups divided by ResNet-50 based on pre-training of the ImageNet dataset, showing different results for the training cohort and testing cohort; (B) patient subgroups divided by ResNet-50 based on pre-training of the NCT-CRC-HE dataset, showing different results for the training cohort and testing cohort; (C) patient subgroups divided by ResNet-50 based on pre-training of the TCGA-BLCA dataset, showing different results for the training cohort and testing cohort. ResNet-50 delineated patient subgroups with different results for the training cohort and testing cohort.

Survival analysis can visualize the whole process of an event and identify the key factors that affect the survival time of a sample by creating multiple survival curves. The SurvGRN model predicted risk values to categorize patients into low-risk and high-risk groups, and the corresponding survival curves for the three datasets are shown in Figure 5. Across both the training and test sets, the *P*-values of the survival curves for the risk groups were less than 0.05, indicating statistical significance. Notably, the survival curves based on the TCGA-BLCA pre-trained model exhibited stronger statistical significance compared to other methods.

**Figure 6.**
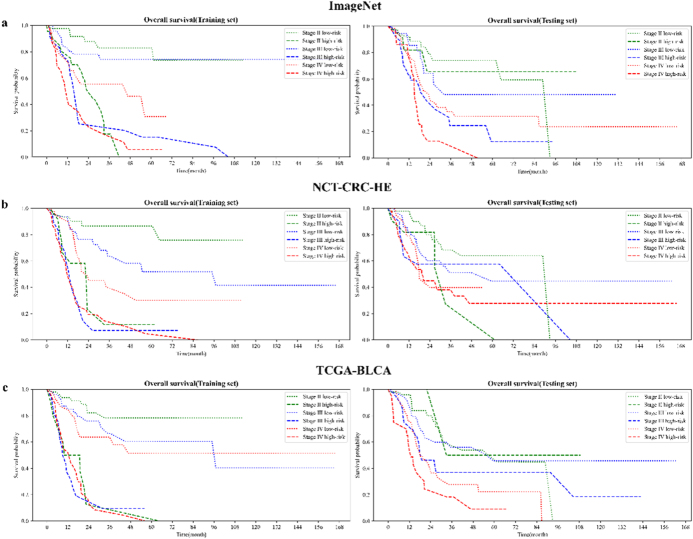
Classification of bladder patients according to model-predicted survival risk values and the TNM staging system. (A) Patient subgroups divided by ResNet-50 based on pre-training using the ImageNet dataset, showing different results for the training cohort and testing cohort; (B) patient subgroups divided by ResNet-50 based on pre-training using the Human Colorectal Cancer and Healthy Tissue image dataset, showing different results for the training cohort and testing cohort; (C) patient subgroups divided by ResNet-50 based on pre-training using the Bladder Cancer image dataset, showing different results for the training cohort and testing cohort. ResNet-50 delineated patient subgroups with different results for the training cohort and testing cohort.

To further validate the performance of the model, we subdivided the TNM staging system into high-risk and low-risk groups classified using the model and plotted the survival curves, as shown in Figure 6.

Table 6 presents the results for subgroups divided based on the combination of the method of pre-training the model with TNM staging in each dataset. Based on the survival curves plotted for this subgroup, we assessed the performance of the pre-trained model in different datasets by counting the number of significant Kaplan–Meier (K–M) curve pairs. There are nine pairs of significant K–M curves in the training set and eight pairs in the test set based on the ImageNet dataset; nine pairs of significant K–M curves in the training set and three pairs in the test set based on the NCT-CRC-HE dataset; nine pairs of significant K–M curves in the training set and three pairs in the test set based on the TCGA-BLCA dataset; and 10 pairs in the training set and six pairs in the test set based on the TCGA-BLCA dataset. It can be observed that the bladder cancer risk group with the pre-training method utilizing the ImageNet dataset shows stronger significance, followed by the pre-training method utilizing the TCGA-BLCA dataset, and least by the pre-training method utilizing the NCT-CRC-HE dataset.

**Table 6:** TNM staging K–M curve *P*-value table.

K–M1	K–M2	*P*-value					
		ImageNet		NCT-CRC-HE		TCGA-BLCA	
		Train set	Test set	Train set	Test set	Train set	Test set
II-low	II-high	*P<0.0001	0.958	*P<0.0001	0.055	*P<0.0001	0.316
II-low	III-low	0.317	0.343	0.060	0.128	0.115	0.664
II-low	III-high	*P<0.0001	*P<0.0001	*P<0.0001	0.170	*P<0.0001	0.106
II-low	IV-low	*P<0.0001	*0.006	*P<0.0001	*0.012	*0.023	*P<0.0001
II-low	IV-high	*P<0.0001	*P<0.0001	*P<0.0001	*P<0.0001	*P<0.0001	*P<0.0001
II-high	III-low	*P<0.0001	0.685	*0.004	0.632	*P<0.0001	0.332
II-high	III-high	0.558	*0.044	0.204	0.928	0.507	0.092
II-high	IV-low	0.182	0.135	0.199	0.578	*0.003	*0.047
II-high	IV-high	0.266	*0.005	0.297	0.495	0.487	*0.022
III-low	III-high	*P<0.0001	*0.024	*P<0.0001	0.469	*P<0.0001	0.110
III-low	IV-low	*0.015	0.089	0.068	0.333	0.515	*0.009
III-low	IV-high	*P<0.0001	*P<0.0001	*P<0.0001	*0.041	*P<0.0001	*P<0.0001
III-high	IV-low	0.098	0.335	*P<0.0001	0.888	*P<0.0001	0.502
III-high	IV-high	0.282	0.155	0.965	0.373	0.721	0.104
IV-low	IV-high	*0.006	*0.008	*P<0.0001	0.515	*P<0.0001	0.089

^*^ indicates that the *P*-value is statistically significant, suggesting a meaningful difference between the two.

These results indicate that the pre-training method using the TCGA-BLCA dataset was more effective in survival prediction as well as in classifying high-risk and low-risk patients, whereas the method pre-trained using the ImageNet dataset was more effective in combining the TNM staging system to further classify high-risk and low-risk patients. It suggests that the method of pre-training using the TCGA-BLCA dataset can improve the performance of the SurvGRN model in survival prediction and survival analysis tasks.

## Discussion

In this paper, we propose SurvGRN, a survival analysis model for bladder cancer patients that integrates clinical data, transcriptomics information, and digital pathology slide information. The model is designed to assess survival risk values of patients and classify them into high-risk cohorts and low-risk cohorts. Additionally, SurvGRN can be combined with the TNM staging system to further classify patients. A key feature of the model is its ability to quantify the influence of various factors on predictions, enabling the identification of critical factors in assessing survival outcomes. These insights allow clinical researchers to focus on specific mechanisms, potentially optimizing treatment plans for bladder cancer patients. Com-parison experiments with state-of-the-art survival analysis methods show that SurvGRN outperforms competing approaches, highlighting the advantages of fusing multi-omics data for survival analysis as well as the validity of the constructed model structure with the pre-training of the ResNet-50 method based on the TCGA-BLCA dataset.

Although the work in this paper is compelling in terms of experimental results, there are some limitations. Due to the limitation of arithmetic power, the analysis of digital pathology slide data was restricted to a single size level, leaving other levels of information unexamined. Additionally, the survival analysis model incorporates a limited set of information, which may fall short of capturing the complexity of the biological systems involved.

As digital pathology continues to evolve alongside advancements in artificial intelligence and information technology, there is a growing need for precise treatments that integrate digital platforms with traditional clinical methods. While this study addresses some current challenges, several directions for future research are proposed to overcome existing limitations and improve upon the current work:

(1) Improved Feature Extraction from Digital Pathology Slides: This paper utilized the pre-trained ResNet-50 model to extract features, but given the high complexity and precision of digital pathology slides, the extracted features represent only a fraction of the available information. Future studies could focus on extracting features across multiple layers of digital pathology slides. For instance, constructing image datasets from different layers, training models to extract layer-specific features, and combining these features in a pyramid structure could significantly advance prediction accuracy.

(2) Building More Comprehensive Survival Analysis Models: While this study successfully integrates clinical, transcriptomics, and digital pathology data for survival risk assessment, it is clear that this information is insufficient to fully represent the complexity of biological systems. Future research could adopt a multi-omics approach by incorporating additional layers of information, such as genomics, microbiomics, epigenomics, metabolomics, proteomics, and transcriptomics. Key questions to explore include how to use deep learning methods effectively for extracting features from individual omics layers and how to combine features across multiple omics layers for robust survival analysis. By addressing these challenges, future studies have the potential to further advance survival analysis models and contribute to more accurate and personalized treatment strategies for bladder cancer patients.

## Method

### Gated residual network

The GRN is obtained by combining and transforming the Gated Linear Unit (GLU) with the Residual Network structure, enabling it to effectively process time-series data while mitigating gradient explosion.

Assuming that the input of GRN is g ∈ Rm, where g is a one-dimensional vector and m is the number of eigenvectors of the vector; and the optional additional input of the model is e ∈ Rm, which is also a one-dimensional vector, the computation process of GRN is as follows:

(1) $${D_{out_{e}}}^{T}=ELU(W_{De_{1}}.g^{T}+W_{De_{2}}.e^{T}+b_{De})$$

(2) $${D_{out_{2}}}^{T}=W_{D2}.{D_{out_{e}}}^{T}+b_{D2}$$

(3) $${D_{out_{GLU}}}^{T}=\sigma \left(W_{GLU_{1}}.{D_{out_{2}}}^{T}+b_{GLU_{1}}\right)\left(W_{GLU_{2}}.{D_{out_{2}}}^{T}+b_{GLU_{2}}\right)$$

(4) $$GRN\left(g,e\right)=LayerNorm\left(g+D_{out_{GLU}}\right)$$

First, the input feature vector g and the additional input vector e, after passing through the first Dense layer and going through the ELU activation function, outputs $D_{out_{e}}$, where $W_{De_{1}},W_{De_{2}}\in R^{d}$, $b_{De}\in R^{1}$;

second, the $D_{out_{e}}$ feature output from the first layer, after passing through the second Dense layer, is output $D_{out_{2}}$, where $W_{D2}\in R^{d},b_{D2}\in R^{1}$; third, the output of the second layer, $D_{out_{2}}$, after the nonlinear transformation of the GLU network, the output is $D_{out_{GLU}}$, where $W_{GLU1},W_{GLU2}\in R^{d},b_{GLU1},b_{GLU2}\in R^{1}$;

Finally, the feature $D_{out_{GLU}}$ after the third optimization of the GLU network will be added with the input feature vector g and normalized by the feature layer, and the output is the output of the GRN structure.

### Static variable extractor

Static variable extractor (SVS) is commonly used to process static data features; in addition, it is able to generate the relative degree of influence of each input vector on the output values. The so-called static data refer to data that do not change over time, such as the gender of the patient, the treatment mode, and current gene expression. SVS is a network structure composed of multiple GRNs, and each input feature value will be transformed into a feature by the corresponding GRNs. The GRNs will also be utilized to calculate the degree of contribution of each feature to the output value.

Suppose, the input features of SVS are $S=\left\{s_{1},\ldots ,s_{n}\right\},s_{i}\in R^{m}$, which means that there are *n* features input to the SVS model, and the dimension of each feature vector is *m*. Similarly each input feature may have additional input features $e_{i}\in R^{m}$. The computation procedure of SVS is as follows:

(5) $$g\;Out_{i}=GRN_{i}\left(s_{i},e_{i}\right)$$

(6) $$g\;Out=concat\left(\left[Out_{1},\ldots ,Out_{n}\right]\right)$$

(7) $$a=softmax\left(\left[aGRN_{1}\left(s_{1},e_{1}\right),\ldots ,aGRN_{1}\left(s_{n},e_{n}\right)\right]\right)$$

(8) Out= agOut

First, multiple feature vectors of the input model are operated by the GRN network structure to output the corresponding feature values, where $GRN_{i}$ is the GRN feature converter corresponding to the *i* feature value. $s_{i}$ is the input vector of the *i* feature, $e_{i}$ is the additional input feature vector of the *i* feature, $gOut_{i}$ is the output of the *i* feature after gating residuals operation, and gOut is the output of the *n* features after the GRN structure operation and superposition.

Next, the same input features $s_{i}$ and additional $e_{i}$ are passed through their corresponding GRNs once again. The resulting feature values are super imposed together to perform softmax operation, and finally the coefficient value *a* of the *n* features is generated.

Finally, the coefficient values are multiplied by the GRN-transformed features to generate new features.

### Data composition

The survival analysis model of bladder cancer patients, based on multi-feature fusion, incorporates clinical data, transcriptomics information, and digital pathology slide data from a total of 400 patients. The dataset is randomly divided into a training set (50%) and a testing set (50%). Clinical and digital pathology slide data are sourced from the TCGA-BLCA dataset via the publicly available GDC database, which contains the digital pathology slides and the clinical data of the patients. Meanwhile, the transcriptomics information is obtained from the GDC, UCSC Xena (http://xena.ucsc.edu/), cBioPortal (http://www.cbioportal.org/), and The Human Protein Atlas (http://www.proteinatlas.org/) websites.

The clinical information data encompass information on the patient’s survival status (alive or deceased), treatment modality, TNM staging status, diagnosis time, tumor location, and gender. These data includes both numerical and categorical values, with examples of the clinical data provided in Table 7, and a comparison table of the clinical data is shown in Table 8.

**Table 7:** Clinical data example.

case_id	Stage	vital_status	Pharmaceutical therapy, NOS	Radiation therapy, NOS
PATIENT_0	III	Alive	No	No
PATIENT_1	IV	Dead	No	Yes
PATIENT_400	III	Alive	Not reported	Not reported
case_id	sex	stage_M	age_at_diagnosis	tissue_or_organ_of_origin
PATIENT_0	Male	MX	27 826	Bladder, NOS
PATIENT_1	Female	M0	22 314	Posterior wall of bladder
PATIENT_400	Male	M0	N0	T3a

**Table 8:** Clinical data abbreviations comparison table.

Abbreviation	Description
case_id	Patient’s medical record number
stage	TNM staging result
vital_status	Patient’s vital status
Pharmaceutical therapy, NOS	Whether pharmaceutical therapy is administered
Radiation therapy, NOS	Whether radiation therapy is administered
Sex	Patient’s sex
stage_M	Metastasis status in TNM staging
age_at_diagnosis	Age at the time of diagnosis
tissue_or_organ_of_origin	Information on the location of the tumor

The transcriptomics information featured the expression of proto-oncogenes such as FGFR3, E2F3, and FGFR1; the expression of oncogenes such as RUNX3, ARID1A, and CDKN2A; and the expression of typing factors such as PPARG, UPK3A, and KRT5, as shown in Table 9.

**Table 9:** Transcriptomics information example.

Case_id	FGFR3	E2F3	FGFR1	EGFR	KRT7	RUNX3
PATIENT_0	543.8596	1083.333	446.8811	1081.35	39 170.57	688.1092
PATIENT_1	4065.704	982.7239	286.6737	723.0064	119.9517	550.7876
PATIENT_400	50.3846	821.3131	255.044	103.8903	43 621.89	24.0776
case_id	ARID1A	CDKN2A	PTEN	TP53	EP300	KDM6A
PATIENT_0	2387.427	3939.571	1644.737	3733.431	736.8421	499.5127
PATIENT_1	1547.268	3426.878	1530.21	1386.598	1456.478	811.6001
PATIENT_400	4006.242	8855.2	845.8366	2321.703	1340.765	195.7418

The digital pathology slide information for the patient’s digital pathology slide of bladder cancer requires preprocessing, where the slices are segmented into blocks. These blocks are then preprocessed using the pre-trained ResNet-50 model, and the features of the extracted multiple blocks are spliced together as the features of the pathology slide. The usual method is to use the ImageNet dataset to pre-train the ResNet-50 model to extract features from digital pathology slides. In order to explore the effect of training models from different datasets on the final prediction, this paper adds a dataset using human colorectal cancer and healthy tissue image datasets, as well as a dataset of bladder cancer pathological slide plots produced in this paper. The latter dataset was created from 50 TNM-staged II-IV sections, which were segmented into 224 × 224 blocks that were carefully selected and categorized, including 32 740 images. In subsequent experiments, the image features extracted using ResNet-50 trained on these three datasets are labeled ImageNet, NCT-CRC-HE, and TCGA-BLCA, respectively, and are used for survival analysis prediction.

### Experimental index

In this paper, the consistency index^[^53^]^ (C-index) is employed to evaluate the performance of the survival analysis model for bladder cancer patients.

The C-index is a metric used to evaluate the performance of the model, which indicates the ratio of the consistency between the predicted results of the model for all samples and the results of the actual samples. Taking survival analysis as an example, if the predicted survival time of the patients is longer than that of the actual patients, it means that the predicted results are consistent with the actual results.

(9) $$C-index=\frac{Predictive\;Consistency\;Sample\;Size}{Number\;of\;all\;samples}$$

### Loss function

The function used to conduct the bladder cancer survival analysis experiment is the Cox proportional risk function. The basic concept behind the Cox proportional hazard model is to construct a future risk function based on the baseline risk function and the factors affecting the risk value, which are calculated as follows:

(10) $$F\left(C,t\right)=F_{0}\left(t\right)e^{aC}$$

The objective function for the Cox proportional analysis function is provided in DeepSurv^[^54^]^ as:

(11) $$L=\underset{p:E_{p}=1}{\prod }\frac{e^{H_{0}\left(c_{p}\right)}}{\sum_{q\in R\left(T_{p}\right)}e^{H_{0}\left(c_{q}\right)}}$$

where $H_{0}\left(c\right)=b^{T}c$ denotes the proportional risk value as a function of the risk factor, $R\left(T_{p}\right)$ denotes all the risk events that occurred in this patient before the termination event occurred, and $p:E_{p}=1$ indicates the set of all patients in this sample that had a risk event.

The loss function corresponding to this objective function is:

(12) $$L^{\prime }=l=-\log L=-\sum_{p:E_{p}=1}\left[H_{0}\left(C_{P}\right)-\log\sum_{q\in R\left(T_{P}\right)}e^{H_{0}\left(C_{q}\right)}\right]$$

## Data Availability

This article is available upon reasonable request. TCGA-BLCA data and corresponding labels are available from the GDC Data Commons website (https://portal.gdc.cancer.gov), and NCT-CRC-HE human colorectal cancer HE-stained tissue sections and corresponding labels are available through the publicly available Zenodo website (10.5281/zenodo.1214456).

## Conclusion

In this study, we proposed a novel interpretable deep learning framework, SurvGRN, for survival analysis in bladder cancer patients by integrating clinical information, transcriptomic information, and digital pathology slide information. By incorporating a multi-instance learning framework with gated residual networks and LSTM structures, SurvGRN effectively enhanced multimodal feature extraction and survival risk prediction. On a multi-feature dataset containing 400 bladder cancer patients, SurvGRN achieved superior prognostic performance compared with existing methods, including DeepMISL, Patch-GCN, and Surformer. Ablation studies further demonstrated the effectiveness of multimodal feature fusion and key model components in improving prediction performance. Moreover, SurvGRN identified important prognostic biomarkers and regulatory pathways associated with clinical outcomes, including ARID1A, KDM6A, CDKN2A, and PTEN, providing valuable insights for personalized prognosis evaluation and precision treatment in bladder cancer patients.
